# Efficacy and safety of a self‐expandable stent‐based endobiliary radiofrequency ablation: A preclinical study

**DOI:** 10.1002/btm2.70195

**Published:** 2026-11-05

**Authors:** Dong‐Sung Won, Yubeen Park, Ji Won Kim, Jinsu An, Hyung‐Sik Kim, Se‐Chul Lee, Youngjae Lee, Dongwook Oh, Jung‐Hoon Park

**Affiliations:** ^1^ Biomedical Engineering Research Center, Asan Institute for Life Sciences Asan Medical Center Seoul Republic of Korea; ^2^ Department of Convergence Medicine Asan Medical Center, University of Ulsan College of Medicine Seoul Republic of Korea; ^3^ Department of Biomedical Engineering, School of ICT Convergence Engineering, College of Science & Technology Konkuk University Chungju‐si Chungcheongbuk‐do Republic of Korea; ^4^ Department of Mechatronics Engineering, School of ICT Convergence Engineering, College of Science & Technology Konkuk University Chungju‐si Chungcheongbuk‐do Republic of Korea; ^5^ OSY Med Inc. Yongin Republic of Korea; ^6^ Department of Gastroenterology Asan Medical Center, University of Ulsan College of Medicine Seoul Republic of Korea

**Keywords:** common bile duct, malignant biliary obstruction, radiofrequency ablation, self‐expandable stent electrode

## Abstract

The temperature‐controlled, bipolar self‐expandable stent (SES)‐based radiofrequency ablation (RFA) system represents a novel endobiliary RFA approach; however, its efficacy and safety must be verified before clinical application. The study aimed to investigate the efficacy and safety of SES–RFA in a porcine common bile duct (CBD) model. Ablation ranges were investigated using ex vivo porcine liver according to radiofrequency energy and time. For the in vivo study, radiofrequency parameters were set at 20 W for a 20‐mm electrode and 25 W for a 25‐mm electrode at 70°C for 120 s. Twelve pigs underwent endoscopic‐guided SES–RFA using either electrode (*n* = 6 each). Animals were randomly euthanized at 24 h or 2 weeks after SES–RFA (*n* = 3 per group). The RFA‐treated CBDs were evaluated using cholangiography, cholangioscopy, hematology, histology, and immunohistochemistry. The SES–RFA system was successfully deployed and retrieved through‐the‐scope without device failure. All animals survived until the study endpoint without RFA‐related adverse events, including ductal perforation, contrast leakage, or hematoma. Hepatobiliary levels of alkaline phosphatase, aspartate aminotransferase, alanine aminotransferase, and gamma‐glutamyl transferase were significantly higher at 2 weeks than at 24 h post‐SES–RFA (all *p*< 0.05). Ablation ranges and degree of mucosal damage, including collagen and TUNEL‐positive deposition, were significantly increased at 2 weeks than at 24 h (all *p*< 0.05). The temperature‐controlled bipolar SES–RFA system effectively and safely produced circumferential coagulative necrosis without ductal perforation in the porcine CBD. The system may be used for managing unresectable malignant biliary obstruction in anatomically challenging regions where conventional RFA is difficult to use.

**Translational Impact Statement**

EB‐RFA is a promising therapeutic option for MBO; however, conventional catheter‐based RFA often yields inhomogeneous ablation, leading to unpredictable efficacy and safety concerns. Herein, we developed a temperature‐controlled SES–RFA system optimized for clinical application. This novel SES–RFA system achieved reproducible, uniform circumferential ablation without perforation and was easily delivered via a standard through‐the‐scope approach. Our findings suggest that the SES–RFA system represents a promising therapeutic alternative to conventional EB‐RFA, particularly for non‐tight or anatomically complex strictures, to improve clinical outcomes in patients with unresectable MBO.

## 1 INTRODUCTION

Endobiliary radiofrequency ablation (EB‐RFA) is a minimally invasive therapeutic option for unresectable malignant biliary obstruction (MBO).^1^, ^2^ In addition to providing local control of the tumor after biliary stent placement, it prolongs stent patency and patient survival.^1^, ^2^, ^3^ Over the past few years, two endobiliary RFA catheters, including power‐controlled Habib™ EndoHPB (Boston Scientific, Marlborough, USA) and temperature‐controlled ELRA™ catheter (Starmed, Goyang, Korea), have become commercially available.^4^, ^5^ Despite their potential, conventional bipolar catheter‐based RFA with two or four electrodes often produces inhomogeneous ablation zones and incomplete ablation because of inadequate contact between the electrode and the target tissues.^6^, ^7^, ^8^ Regions in direct contact with the electrodes exhibit a greater degree of ablation than non‐contact regions, resulting in uneven ablation depth and potential adverse events because of excessive tissue damage.^9^ Furthermore, the outcomes of EB‐RFA are affected by lesion characteristics, including stricture length, ductal curvature, and stricture morphology.^8^, ^10^ These factors can make the efficacy unpredictable, particularly in cases involving curved lesion or short, soft, and non‐tight strictures.^5^, ^11^ These limitations of current EB‐RFA catheters significantly reduce the therapeutic outcomes and safety of EB‐RFA in patients with unresectable MBO.^12^

Recently, a temperature‐controlled self‐expandable stent (SES)‐based endobiliary RFA (SES–RFA) system has been introduced.^12^, ^13^ This novel bipolar SES–RFA system successfully generates outstanding circumferential coagulative necrosis in the porcine bile duct, and the ablation ranges can be easily controlled by simple adjustment of RF power and ablation time.^12^ Although the preliminary results of the SES–RFA system have been reported, additional studies are required to confirm the long‐term efficacy and safety of the system before clinical trials. Therefore, we developed a final prototype of the SES–RFA system optimized for clinical applications. The prototype was optimized by modifying electrode specifications and handle design to facilitate endobiliary RFA via the endoscopic working channel. This system is currently under review by the Korean Ministry of Food and Drug Safety. This preclinical study aimed to investigate the efficacy and safety of the SES–RFA system in a porcine model.

## 2 MATERIALS AND METHODS

### 2.1 Preparation of the SES–RFA system

The SES electrode was fabricated by braiding 64 nitinol wires (0.09 mm thick). When fully expanded, each SES electrode was 8 mm in diameter and 5 mm in length (S&G Biotech Co., Ltd., Yongin, Korea). Two types of bipolar SES electrodes were prepared according to the distance between the first and second electrodes, measuring 20 and 25 mm (Figure 1a). The SES electrode was loaded onto an 8‐Fr delivery system with a usable length of 1800 mm, ensuring compatibility with the endoscope. The delivery system included an insulated outer tube, an inner tube with a guide olive tip, a handle for deployment and retrieval of the SES electrode, and a connector to the RFA generator. The handle was designed as a pistol type and consisted of a trigger and a directional slide. When the directional slide was pulled back, the SES electrodes fully expanded. When the trigger was pulled on the handle, the expanded SES electrodes collapsed to recapture the electrode into the delivery system. When the directional slide was pushed forward, the collapsed electrodes were recaptured into the delivery system for removal post‐RFA (Figure 1b and Supplementary Movie 1).

**FIGURE 1:**
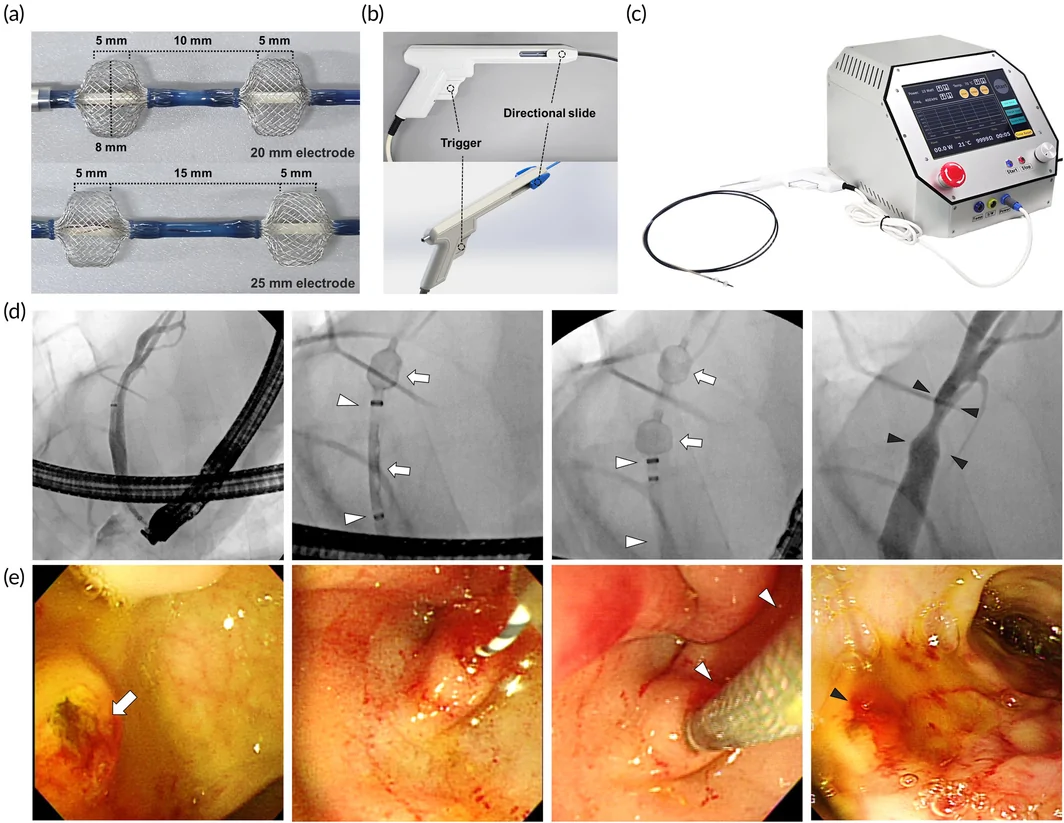
Temperature‐controlled self‐expandable stent (SES)‐based radiofrequency ablation (RFA) system for endobiliary RFA in the porcine bile duct. (a) The SES electrode is composed of an anode and a cathode, and each SES electrode was 5 mm in length and 8 mm in diameter. Two lengths of electrodes were used in this study. (b) The delivery handle consists of a directional slide and delivery trigger for controlled deployment and recapture of the SES electrode. (c) The RFA generator allows easy controls and real‐time monitoring of radiofrequency parameters such as power, frequency, impedance, and temperature. (d) Fluoroscopic images obtained during the procedure show pre‐cholangiography status, expanded SES electrodes (*arrows*) with the delivery system (*arrowheads*), and stricture formation (*black arrowheads*) on post‐cholangiography. (e) Endoscopic images obtained during the procedure show the ampulla of Vater (*arrow*), the inserted SES delivery system (*arrowheads*) via guidewire, and the ampulla of Vater (*black arrowhead*) post‐RFA.

An optimized RFA generator (Konkuk University, Chungju, Korea) for SES electrodes was used to deliver energy, with output power (5–80 W), frequency (10–500 kHz), temperature (5–120°C), impedance (10–1200 Ω), and ablation time. Impedance and temperature changes were monitored in real time during RFA (Figure 1c).

### 2.2 Ablation ranges of the SES–RFA system in the porcine liver

Extracted porcine livers were used to evaluate ablation ranges under different ablation power and time before in vivo study. Livers were cut into a rectangular parallelepiped shape, and an SES electrode was placed at the center using a stylet.^14^ Ablation was performed at two RF output powers (20 and 25 W) and ablation times (120 and 180 s) using two types of SES electrodes (20 and 25 mm), with frequency set at 500 kHz and temperature at 70°C. The ablated livers were sectioned along the long axis of the electrode and photographed to evaluate ablation range (ablation length and depth). Ablation length was measured as the maximum length ablated along the SES axis, and ablation depth was measured as the maximum length ablated along the vertical SES axis. All ex vivo studies were repeated five times to determine statistical reproducibility.

### 2.3 Animal study design

This study was approved by the Institutional Animal Care and Use Committee of the Asan Institute for Life Sciences (Seoul, Korea) (2022‐12‐024) and conformed to the US National Institutes of Health guidelines for the humane handling of laboratory animals. Twelve male Yorkshire pigs (aged 4 months; mean body weight; 39.2 kg; range, 37.5–40.5 kg) (International Animal Experiment Center, Pocheon, Korea) were included in the study. RFA parameters were fixed at 500 kHz and 70°C for 120 s, with RF power set to 20 W for the 20‐mm electrode and 25 W for the 25‐mm electrode according to the ex vivo experimental findings. The pigs were randomly allocated to the 20 or 25‐mm electrode group (*n* = 6 per group) and then to euthanasia at 24 h or 2 weeks post‐RFA (*n* = 3 per time point) using a computer‐generated random number sequence. Group allocation was performed by an investigator who was not involved in the procedures or outcome assessments, and cholangiographic measurements and histological evaluations were performed by investigators blinded to the group allocation. The sample size was determined based on our previous preclinical studies without a formal power calculation, in accordance with the 3Rs principle. No animals were excluded from the analysis. All pigs underwent endobiliary RFA and were euthanized at the allocated time point by injection of potassium chloride (JW Pharm, Seoul, Korea) via the ear vein under deep anesthesia. Antibiotics (Gentamicin, 7 mg/kg; Shin Poong Pharm Ltd., Seoul, Korea) and analgesics (Keromin, ketorolac 1 mg/kg; Hana Pharm Ltd., Seoul, Korea) were routinely administered for 3 days after the procedures.

### 2.4 Endobiliary RFA procedure using the SES–RFA system in the porcine common bile duct

Under general anesthesia, the common bile duct (CBD) cannulation was performed using a standard sphincterotome (CleverCut 3VTM; Olympus Medical Systems, Tokyo, Japan) and a 0.035‐inch guidewire (Revowave; Olympus) under endoscopic guidance (TJF‐260V; Olympus). A pre‐cholangiogram was obtained to locate the CBD for endobiliary RFA under fluoroscopic guidance (OEC Elite CFD; GE HealthCare, Chicago, IL, USA). The SES electrode was advanced through the guidewire into the targeted CBD under combined endoscopic and fluoroscopic guidance, and the inserted SES were fully expanded (Figure 1d,e). RFA procedure was performed using two different lengths of the SES electrodes. The expanded SES electrode was recaptured into the delivery system and then removed immediately post‐RFA. A post‐cholangiogram confirmed the presence of ductal perforation or stricture formation. Temperature was continuously monitored and recorded throughout the procedure.

### 2.5 Hematological examination

Blood samples from the internal jugular vein were collected before the procedure and before euthanization of pigs. The collected blood samples were analyzed to determine white blood cell (WBC) and platelets (PLT) counts and hemoglobin (Hb), hematocrit (HCT), total bilirubin, alkaline phosphatase (ALP), aspartate aminotransferase (AST), alanine aminotransferase (ALT), and gamma‐glutamyl transferase (GGT) levels to evaluate the degrees of inflammation and hepatobiliary functions.

### 2.6 Cholangiographic examination

Cholangiography was performed before and immediately after the procedure, as well as at 24 h and 2 weeks after the procedure. The luminal diameter and the length of stricture formation of the RF‐ablated CBDs were measured on each cholangiogram using Radiant software (Medixant, Poznan, Poland). The stricture length was defined as the narrowed luminal segment along the CBD axis. The luminal diameter was defined as the inner diameter of the RF‐ablated CBD.

### 2.7 Cholangioscopic examination

Cholangioscopic examination was performed to evaluate the degrees of stricture formation and mucosal injuries before euthanization of pigs. A 2.9‐mm‐diameter laryngoscope (Karl Storz, Tuttlingen, Germany) was inserted through the exposed second portion of the duodenum and advanced via the ampulla of Vater to evaluate the intraluminal bile duct. The degree of mucosal injuries in the RF‐ablated CBDs was examined.

### 2.8 Histological examination

The entire liver, duodenum, and bile duct were surgically extracted and fixed in 10% neutral buffered formalin for 48 h. RFA‐treated CBDs were examined to assess macroscopic ablation length and were longitudinally sectioned (Supplementary Figure 1). The samples were embedded in a paraffin block for hematoxylin and eosin (H&E) and Masson's trichrome (MT) staining. Five histological sections were obtained from each animal (*n* = 15 sections per group), and the values from each section were used for statistical analysis. Microscopic ablation length and depth were determined by identifying coagulative necrosis at the basement membrane of the normal epithelium and the deepest extent of necrotic inflammation.^15^, ^16^ Inflammatory cell infiltration in H&E‐stained sections was subjectively determined in accordance with diffuse infiltration of macrophages as follows: 1 = mild; 2 = mild to moderate; 3 = moderate; 4 = moderate to severe; and 5 = severe. The degree of collagen deposition in MT‐stained sections was subjectively determined as follows: 1 = mild; 2 = mild to moderate; 3 = moderate; 4 = moderate to severe; and 5 = severe.

### 2.9 Immunohistochemistry

Immunohistochemistry was performed using terminal deoxynucleotidyl transferase‐mediated dUTP nick‐end labeling (TUNEL) and heat shock protein 70 (HSP 70) to evaluate the extent of cell death and the presence of heat shock protein following endobiliary RFA. The extents of TUNEL and HSP 70‐positive deposition were subjectively determined as follows: 1 = mild; 2 = mild to moderate; 3 = moderate; 4 = moderate to severe; and 5 = severe. Histological examinations were conducted using a digital slide scanner (Panoramic 250 FLASH III; 3D Histech Ltd., Budapest, Hungary), and measurements were analyzed using a digital microscope viewer (CaseViewer; 3D Histech).

### 2.10 Statistical analysis

Normally distributed data are presented as mean ± SD and were compared using the two‐way ANOVA with Bonferroni post‐hoc correction. Non‐normally distributed data were compared using the Mann–Whitney *U*‐test. The statistical analysis was performed using SPSS software, version 27.0 (IBM, Chicago, IL, USA). Statistical significance was determined at values of *p*< 0.05.

## 3 RESULTS

### 3.1 Ablation ranges of the SES–RFA system

Representative images of the RF‐ablated liver are shown in Figure 2, and ablation ranges are summarized in Supplementary Table 1. SES–RFA was successfully performed at all RFA parameters without roll‐off in the extracted porcine livers. The uniform circumferential ablation ranges, in depth and length, increased proportionally with RF power and ablation time with both 20 and 25‐mm electrodes (Figure 2a,b). The temperature reached 70°C in 17 s at 20 W and 9 s at 25 W with 20‐mm electrode and in 74 s at 20 W and 50 s at 25 W with 25‐mm electrode, respectively (Figure 2c). Once the temperature reached 70°C, it was maintained consistently for 120 or 180 s.

**FIGURE 2:**
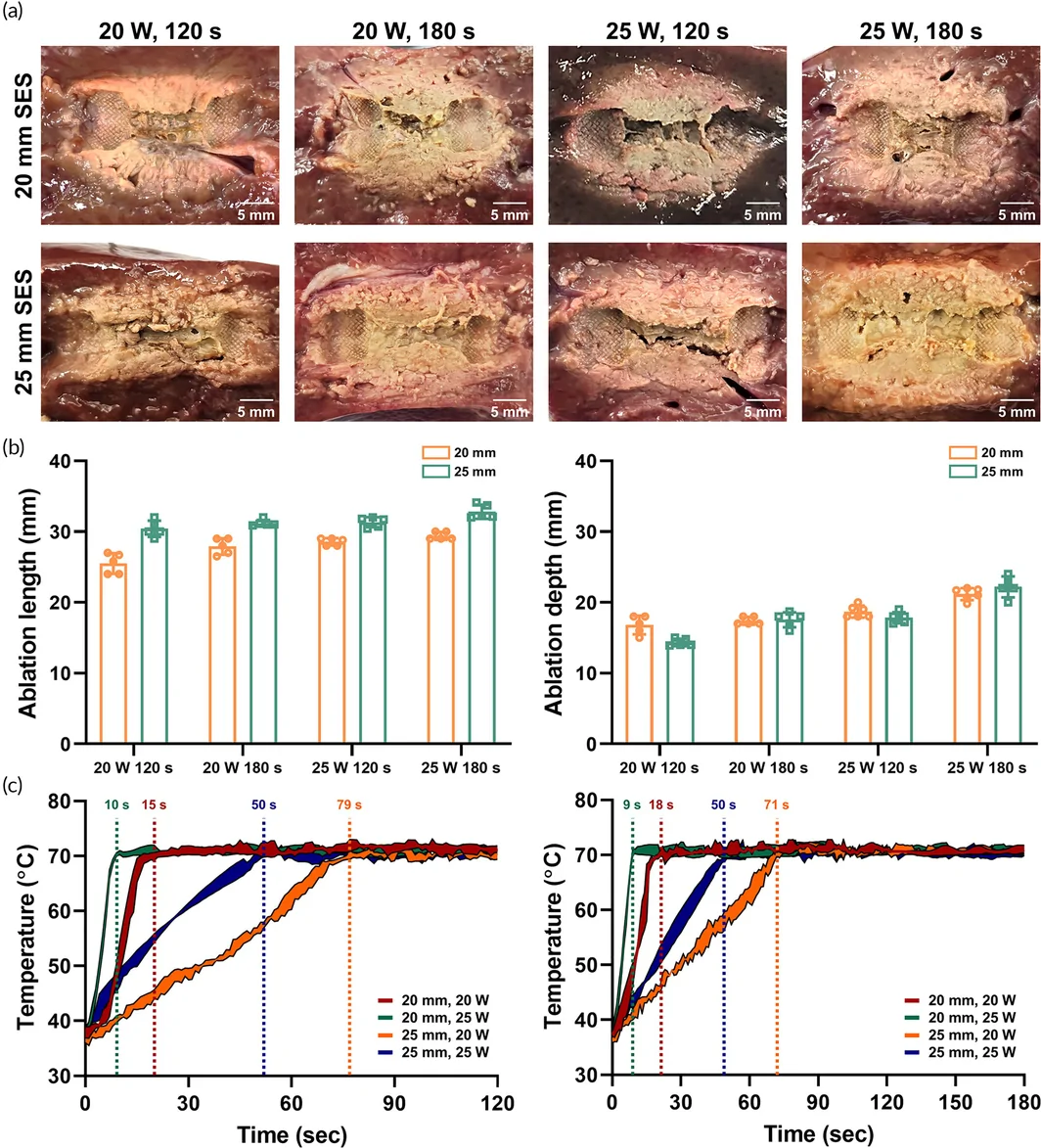
Ex vivo findings after self‐expandable stent (SES)‐based radiofrequency ablation (RFA) in the porcine liver. (a) Representative longitudinal images of the RF‐ablated liver obtained with 20‐ or 25‐mm electrodes. (b) Ablation ranges in depth and length increased proportionally with combined RF power and time using both electrodes. Graphs represent mean ± SD, and dots represent individual data points (*n* = 5 per parameter). (c) The temperature‐controlled SES–RFA system showed different temperature profiles to the target temperature set at 70°C at 20 W (*red and orange dotted lines*) and 25 W (*green and blue dotted lines*) for 20‐ and 25‐mm electrodes. Graphs represent mean ± SD (*n* = 5 per parameter). After reaching the target, the temperature was maintained for 120 and 180 s for 20‐mm and 25‐mm electrodes, respectively.

### 3.2 Procedural outcomes of endobiliary RFA using SES–RFA system

Endobiliary RFA performed using the SES–RFA system with 20 and 25‐mm SES electrodes was technically successful in all pigs. The delivery system of the SES electrodes was successfully deployed into and removed from the target CBD under endoscopic and fluoroscopic guidance without procedure‐related adverse events. Ductal perforations in the RF‐ablated CBDs were not observed in the follow‐up cholangiography following RFA. Significant weight loss, poor oral intake, or clinical signs associated with jaundice were not observed during the follow‐up period. All animals survived until the end of the study without endobiliary RFA‐related adverse events.

### 3.3 Hematological findings

Hematologic findings are summarized in Supplementary Table 2. The mean Hb, HCT, and PLT levels measured before and at 24 h and 2 weeks post‐RFA were within normal ranges without any significant differences (Figure 3d). The mean WBC counts and ALP, AST, and GGT levels gradually increased at 24 h and 2 weeks post‐RFA compared with those before the procedure. The WBC counts and ALP, AST, and GGT levels at 2 weeks were significantly increased beyond the normal ranges (all *p*< 0.001).

**FIGURE 3:**
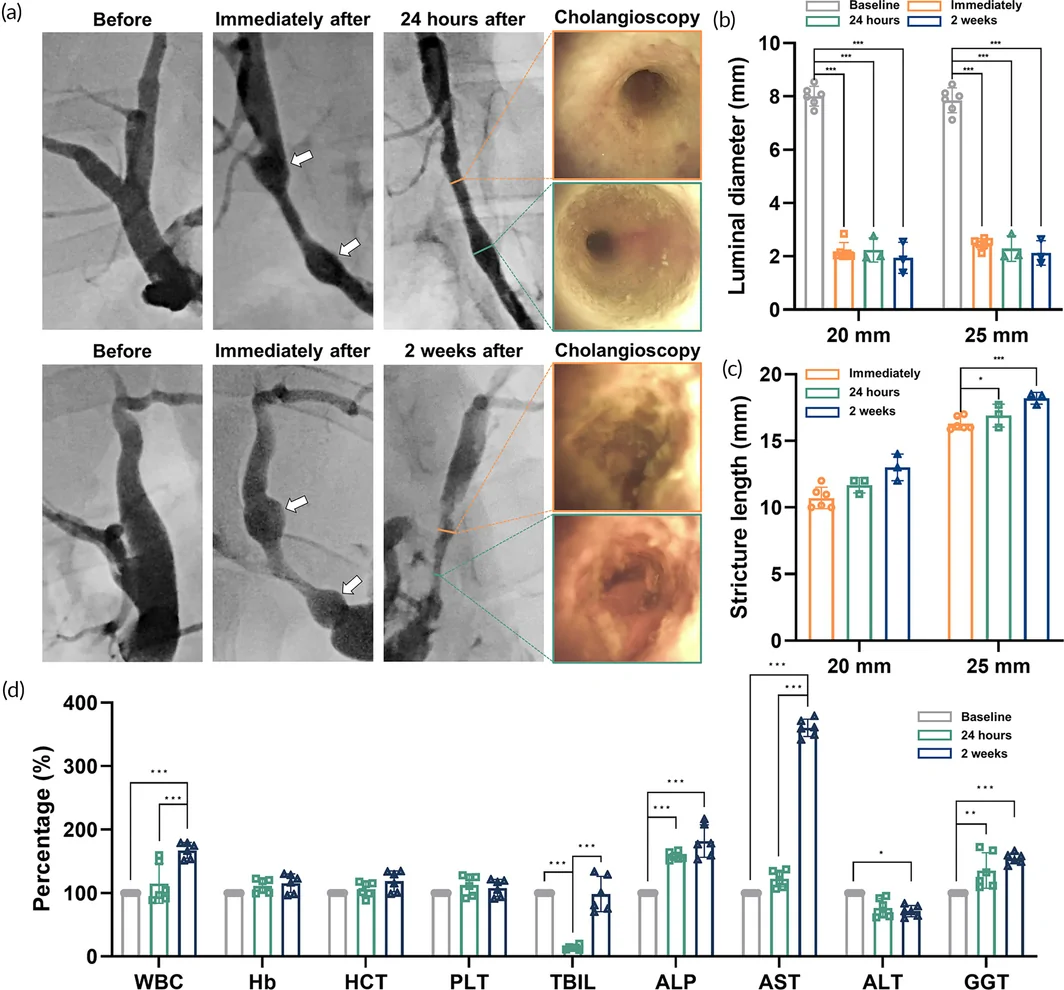
Cholangiographic and cholangioscopic findings in the radiofrequency ablation (RFA)‐treated common bile duct (CBD). (a) Cholangiograms obtained before the procedure, as well as immediately, 24 h, and 2 weeks after the procedure, show significant coagulative necrosis‐induced severe strictures at 24 h and 2 weeks of follow‐up. Similarly, cholangioscopic images show severe mucosal injuries with stricture formation in the RF‐ablated CBD at 24 h and 2 weeks of follow‐up. Graphs show (b) luminal diameter and (c) stricture length of the RF‐ablated CBD generated based on cholangiographic analysis immediately, 24 h, and 2 weeks after the procedure. Graph bars represent mean ± SD, and dots represent individual data points (baseline and immediately after RFA, *n* = 6; 24 h and 2 weeks, *n* = 3 per electrode). (d) Hematology assays revealed changes in complete blood count and hepatobiliary levels. Graph bars represent mean ± SD, and dots represent both 20‐ and 25 mm electrode data (*n* = 6 per date). ALP, alkaline phosphatase; ALT, alanine aminotransferase; AST, aspartate aminotransferase; GGT, gamma‐glutamyl transferase; Hb, hemoglobin; HCT, hematocrit; PLT, platelet; TBIL, total bilirubin; WBC, white blood cells; *< 0.05, **< 0.01, and ***< 0.001.

### 3.4 Cholangiographic and cholangioscopic findings

The representative cholangiographic and cholangioscopic findings are shown in Figure 3a and Supplementary Figure 2. Compared with baseline (8.01 ± 0.38 mm and 7.81 ± 0.51 mm for the 20‐ and 25‐mm electrode, respectively), the luminal diameter of the RF‐ablated CBD decreased significantly immediately after RFA and remained narrowed through 2 weeks with both electrodes (1.99 ± 0.41 mm and 2.19 ± 0.39 mm, respectively; all *p*< 0.001) (Figure 3b). Stricture length progressively increased over time, reaching 13.71 ± 0.71 mm and 18.25 ± 0.31 mm at 2 weeks with the 20‐ and 25‐mm electrodes, respectively. However, this increase was significant only in the 25‐mm electrode (Figure 3c and Supplementary Table 3).

Circumferential mucosal injuries and white‐wish segmental changes with shrinkages caused by coagulative necrosis were observed in the RF‐ablated CBDs at 24 h after the procedure. Definite stricture formation was observed, and the 2.9‐mm‐diameter laryngoscope could not pass through the strictures in two pigs. In the remaining four pigs, the scope could pass through the strictures but with strong resistance. Additionally, abnormal vasculature with neovascularization and easy oozing of blood was observed on the mucosa adjacent to a stricture. At the 2‐week follow‐up, severe strictures with hyperplastic changes and irregular surfaces were observed, and the scope could not pass through the strictures in all pigs. Cholangioscopic examination revealed no adverse events, such as perforation or bleeding, associated with SES–RFA.

### 3.5 Histological findings

Representative histological images are shown in Figure 4 and Supplementary Figure 3, and histological findings are summarized in **S**upplementary Table 4. Microscopic ablation length and depth increased significantly from 24 h to 2 weeks after SES–RFA with both 20‐mm and 25‐mm electrodes (all, *p*< 0.01). However, no significant difference was observed in the mean degree of inflammatory cell infiltration with 20‐mm and 25‐mm electrodes (*p* = 0.454 and *p* = 0.087, respectively) between 24 h and 2 weeks post‐RFA. Furthermore, the mean degree of collagen and TUNEL deposition gradually increased at 2 weeks compared with that at 24 h with 20‐mm and 25‐mm electrodes (all *p*< 0.05), whereas HSP70‐deposition gradually decreased at 2 weeks compared with that at 24 h (all *p*< 0.001).

**FIGURE 4:**
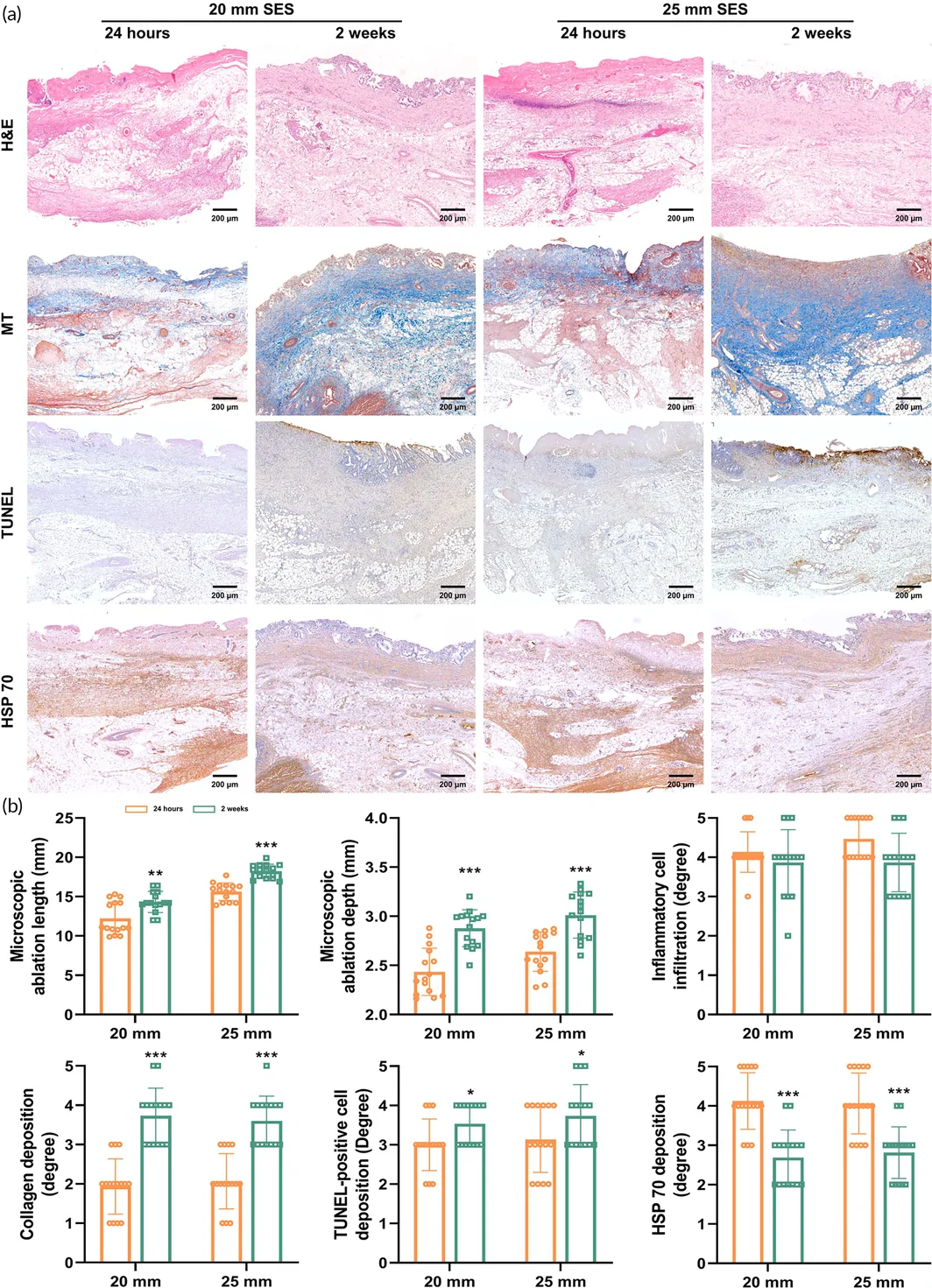
Histologic findings after self‐expandable stent (SES)‐based radiofrequency ablation (RFA) in the porcine common bile duct (CBD). (a) Representative microscopic images of H&E, MT, TUNEL, and HSP70 staining showed significant coagulative necrosis along the CBD wall. (b) Serial histopathological findings for the porcine CBD model 24 h and 2 weeks after the RFA procedure. Graph bars represent mean ± SD, and each dot represents one histological section (five sections per animal from three animals per group; *n* = 15 sections per group). H&E, hematoxylin and eosin; HSP70, heat shock protein 70; MT, Masson's trichrome; TUNEL, terminal deoxynucleotidyl transferase‐mediated dUTP nick end labeling; *< 0.05, **< 0.01, ***< 0.001.

## 4 DISCUSSION

This study demonstrated that the novel temperature‐controlled SES–RFA system was effective and safe in generating uniform circumferential coagulative necrosis without causing ductal perforations during a 2‐week follow‐up period in a porcine CBD model. The SES–RFA system was deliverable through a standard therapeutic duodenoscope using a through‐the‐scope (TTS) approach, and the self‐expanding electrode design has the potential to provide consistent electrode–tissue contact regardless of duct diameter or curvature, although this was not specifically evaluated across anatomical variations in the present study. Ex vivo experiments confirmed that the ablation range increased proportionally with RF power and ablation time, and in vivo evaluation showed predictable microscopic ablation lengths determined by electrode length. Serial biochemical, cholangiographic, cholangioscopic, and histologic assessments demonstrated a time‐dependent progression from mucosal injury to inflammatory infiltration and fibrosis, consistent with controlled thermal ablation and organized tissue remodeling.

Compared with conventional catheter‐based EB‐RFA, the SES–RFA system offers several potential advantages related to its self‐expanding, nitinol‐braided electrode architecture. The self‐expanding design of SES–RFA system may provide more consistent circumferential wall contact than catheter‐based electrodes across different duct diameters and configurations. This addresses the most critical limitation of conventional catheter‐based EB‐RFA, in which uneven electrode–tissue apposition results in heterogeneous ablation depth and occasionally excessive focal thermal injury.^17^ In a recent ex vivo comparative study using a curved endoluminal phantom, the catheter‐based electrode produced curvature‐dependent asymmetric ablation, whereas the stent‐based electrode produced uniform ablation regardless of curvature.^18^ Moreover, while conventional temperature‐controlled catheter systems typically require target temperatures of ≥80°C to generate sufficient ablation depth,^15^, ^19^ the novel SES–RFA system achieved comparable penetration at 70°C, likely due to its stable heat transfer characteristics and uniform contact. The SES–RFA system also enabled a gradual temperature rise and stable impedance, a property attributable to the lower thermal and electrical conductivity of the nickel–titanium alloy electrode, thereby reducing carbonization and minimizing the risk of abrupt roll‐off events.^20^, ^21^

Regarding safety, previous animal studies using temperature‐controlled catheters at target temperatures of 75–80°C reported no perforation or bleeding at 24 h^15^, ^19^ or 4 weeks^22^ after EB‐RFA, whereas bile duct perforation was observed at higher power settings of 80–100 W.^23^ Perforation has been reported with the power‐controlled Habib™ EndoHPB catheter,^5^, ^24^, ^25^ whereas no thermal injury‐related complications, including perforation or hemobilia, were observed in a randomized trial using a temperature‐controlled catheter.^26^ Consistent with these findings, no perforation or hemorrhage occurred in any bile ducts treated with the temperature‐controlled SES–RFA system in the present study, suggesting that temperature regulation may contribute to procedural safety. However, given the small sample size (*n* = 12), the absence of perforation in this study does not exclude the possibility of such complication. Larger studies are therefore warranted to further validate the safety profile of the SES–RFA system.

Furthermore, several important considerations remain. The strong electrode–tissue contact achieved with SES–RFA has the potential to increase localized overheating if thermal parameters are not precisely controlled, as noted in prior SES–RFA liver models.^27^ In addition, SES–RFA generally requires higher RF power compared with catheter‐based electrodes, which emphasizes the necessity of precise temperature‐controlled RFA system to avoid excessive tissue injury.^8^, ^28^, ^29^ The present study supports the reliability of temperature regulation, but confirms the need for careful parameter optimization when treating strictures of variable lengths and configurations.

Our previous studies evaluating SES–RFA suggested that ablation range is influenced by RF power and ablation time; however, these investigations did not characterize how injury evolves over time in vivo.^12^ In the current study, hepatobiliary enzyme profiles demonstrated graded biochemical responses to thermal injury with increases at 2 weeks, consistent with controlled biliary remodeling rather than uncontrolled tissue destruction. Correspondingly, cholangiography and cholangioscopy showed early mucosal disruption and mild luminal narrowing at 24 h, followed by progressive stricture at 2 weeks without perforation or bleeding. Notably, stricture length increased with electrode length, while ablation depth remained stable across electrodes, suggesting that ablation length can be adjusted by selecting the electrode size. This characteristic of SES–RFA may enable lesion‐length–tailored therapy in a single session, reducing the need for multiple overlapping ablations, which are often required when using conventional catheter‐based EB‐RFA devices.^30^, ^31^ In addition, stricture formation after EB‐RFA in the normal bile duct has also been reported with catheter‐based systems. In a long‐term swine study using a temperature‐controlled catheter (80°C, 7–10 W, 120 s), all animals developed biliary strictures at 4 weeks, with a median length of 10.5 mm, accompanied by jaundice and purulent bile. Stricture length was reported to increase in proportion to both the applied power and electrode length.^22^ Although a recent ex vivo study directly compared ablation zones between stent‐based and catheter‐based electrodes,^18^ no in vivo study has compared stricture formation between SES–RFA and catheter‐based EB‐RFA under identical experimental conditions. Moreover, differences in RF parameters, follow‐up duration, and animal models across studies preclude a direct quantitative comparison. The circumferential contact provided by the SES electrode may also facilitate more reproducible and uniform ablation in anatomical challenging setting, such as curved bile ducts, perihilar branching regions, and previously stented ducts; however, these conditions were not evaluated in the present study and warrant further investigation.

Although endobiliary RFA remains a valuable therapeutic option for MBO, the effectiveness of conventional catheter‐based systems is often limited by inconsistent electrode–tissue contact, leading to variable ablation depth and, in some cases, stricture progression or ductal injury.^26^, ^32^, ^33^ These challenges are particularly evident in short, soft, or non‐constrictive strictures, as well as in curved or perihilar segments, where achieving circumferential apposition is difficult^7^, ^9^, ^12^ By providing stable circumferential apposition and temperature‐controlled energy delivery, SES–RFA has the potential to overcome these challenges and expand the applicability of endobiliary ablation, even in anatomically complex regions.

This study has some limitations. The study involved a small sample size and a short follow‐up period, limiting assessment of late stricture maturation or delayed adverse events. Furthermore, anatomical variations of the CBD, including differences in ductal diameter, curvature, and length, were not evaluated because of the limited number of animals. Therefore, the ability of the SES–RFA system to maintain consistent electrode–tissue contact across diverse ductal anatomies, particularly in curved and perihilar segments, should be investigated in further studies. Additionally, healthy porcine CBD differs in composition from malignant human biliary tissue, which may influence thermal penetration and may necessitate adjustment of RF parameters in clinical application.^19^ Adjacent organ safety was not comprehensively evaluated, and biliary stents were not placed post‐RFA, unlike typical clinical workflows. In addition, only male pigs were included in this study. Although sex‐related differences in the acute thermal response of the bile duct remain unclear, sex hormones may influence tissue healing and fibrotic remodeling, potentially affecting stricture formation. Given that MBO occurs in both sexes, future studies should include both male and female animals to assess potential sex‐related differences and improve the translational relevance of the findings.

In conclusion, the temperature‐controlled bipolar SES–RFA system achieved reproducible, uniform circumferential ablation without perforation and was easily delivered through a standard TTS approach. These findings suggest that SES–RFA may provide a promising therapeutic alternative to conventional EB‐RFA, particularly for non‐tight or anatomically complex strictures where catheter‐based EB‐RFA is less effective.

## AUTHOR CONTRIBUTIONS

**Dong‐Sung Won:** Conceptualization; formal analysis; methodology; writing – review and editing; writing – original draft; investigation. **Yubeen Park:** Investigation; writing – original draft; writing – review and editing; formal analysis; methodology. **Ji Won Kim:** Investigation; formal analysis; writing – review and editing; methodology. **Jinsu An:** Investigation; formal analysis; methodology; writing – review and editing. **Hyung‐Sik Kim:** Investigation; formal analysis; methodology; writing – review and editing. **Se‐Chul Lee:** Investigation; formal analysis; methodology; writing – review and editing. **Youngjae Lee:** Investigation; formal analysis; methodology; writing – review and editing. **Dongwook Oh:** Methodology; writing – original draft; writing – review and editing; formal analysis; supervision; validation. **Jung‐Hoon Park:** Conceptualization; methodology; writing – original draft; writing – review and editing; formal analysis; supervision; project administration.

## FUNDING INFORMATION

This study was supported by the Korea Medical Device Development Fund grant funded by the Korean government (the Ministry of Science and ICT, the Ministry of Trade, Industry and Energy, the Ministry of Health and Welfare, Republic of Korea, the Ministry of Food and Drug Safety; project number: RS‐2023‐00238092); by the National Research Foundation of Korea(NRF) grant funded by the Korea government (MSIT) (RS‐2026‐25469139 and RS‐2026‐25475904); by a grant (2026IE0004) from the Asan Institute for Life Sciences, Asan Medical Center, Seoul, Korea.

## CONFLICT OF INTEREST STATEMENT

The authors declare that they have no known competing financial interests or personal relationships that could have appeared to influence the work reported in this paper.

## Supporting information


**Supplementary Figure 1.** Tissue sectioning method for the histological evaluation of tissues ablated with different electrode lengths. The common bile duct (CBD) was unfolded longitudinally, and the unfolded CBD was sectioned longitudinally again (*Orange lines*) to conduct histological examination.
**Supplementary Figure 2**. Representative cholangioscopic evaluation after follow‐up cholangiography. (A) 2.9‐mm laryngoscope was inserted along the segment of the radiofrequency ablated common bile duct (CBD) under fluoroscopic guidance. Cholangioscopic evaluation was performed from the region beyond the first positioned electrode to behind the second positioned electrode in the porcine CBD.
**Supplementary Figure 3**. Macroscopic and microscopic ablation ranges of the self‐expandable stent‐based‐radiofrequency ablation system according to different electrode type in the porcine common bile duct.
**Supplementary Table. 1** Ablation length and depth according to RF parameters and electrode lengths in the porcine liver.
**Supplementary Table 2**. Hematological findings before RFA and after euthanasia.
**Supplementary Table 3**. Cholangiographic findings at baseline and immediately, 24 h, and 2 weeks after RFA in the porcine bile duct.
**Supplementary Table 4**. Histological findings at 24 h and 2 weeks after RFA.

**Supplementary Movie 1.** Procedural steps for deployment and recapture of the self‐expandable stent (SES)‐based endobiliary radiofrequency ablation (SES–RFA) system.

## Data Availability

The data that support the findings of this study are available from the corresponding author upon reasonable request.
