# Supplementary material for: Efficacy and safety of a self‐expandable stent‐based endobiliary radiofrequency ablation: A preclinical study
Source: Bioeng Transl Med. 2026 Nov 5:e70195. Online ahead of print. doi: 10.1002/btm2.70195 (PMC13735443; doi:10.1002/btm2.70195)
Supplement: Supplementary file 1 [file BTM2-9999-e70195-s002.docx]

**Supplementary Figure 1.** Tissue sectioning method for the histological evaluation of tissues ablated with different electrode lengths. The common bile duct (CBD) was unfolded longitudinally, and the unfolded CBD was sectioned longitudinally again (*Orange lines*) to conduct histological examination.

**Supplementary Figure 2.** Representative cholangioscopic evaluation after follow-up cholangiography. A 2.9-mm laryngoscope was inserted along the segment of the radiofrequency ablated common bile duct (CBD) under fluoroscopic guidance. Cholangioscopic evaluation was performed from the region beyond the first positioned electrode to behind the second positioned electrode in the porcine CBD.

**Supplementary Figure 3.** Macroscopic and microscopic ablation ranges of the self-expandable stent-based-radiofrequency ablation system according to different electrode type in the porcine common bile duct.

**Supplementary Table. 1** Ablation length and depth according to RF parameters and electrode lengths in the porcine liver.

| Electrode  length (mm) | Time  (sec) | Ablation length (mm) | | | Ablation depth (mm) | |
| --- | --- | --- | --- | --- | --- | --- |
|  |  | 20 W | 25 W |  | 20 W | 25 W |
| 20 | 120 | 25.43 ± 1.18 | 28.80 ± 0.68 |  | 16.83 ± 1.06 | 18.66 ± 1.59 |
|  | 180 | 27.91 ± 0.93 | 29.33 ± 0.47 |  | 17.41 ± 0.18 | 21.16 ± 3.62 |
| 25 | 120 | 30.16 ± 1.06 | 31.23 ± 1.37 |  | 14.33 ± 1.34 | 17.90 ± 1.08 |
|  | 180 | 31.01 ± 0.55 | 32.86 ± 1.10 |  | 17.56 ± 0.86 | 21.83 ± 1.46 |

Data are presented as mean ± standard deviation.

**Supplementary Table 2.** Hematological findings before RFA and after euthanasia.

| Electrode (L) | Time (T) | WBC (10^3^/μL) | Hb (g/dL) | HCT (%) | PLT (10^3^/μL) | TBIL (mg/dL) | ALP (U/L) | AST (U/L) | ALT (U/L) | GGT (U/L) |
| --- | --- | --- | --- | --- | --- | --- | --- | --- | --- | --- |
| 20 mm | 0 | 14.23 ± 4.36 | 11.35 ± 0.64 | 36.40 ± 0.71 | 279.50 ± 180.31 | 0.15 ± 0.30 | 392.45 ± 221.82^a^ | 27.80 ± 4.81 | 35.95 ± 9.40 | 27.35 ± 6.58 |
|  | 1 | 13.50 ± 1.50 | 12.88 ± 1.10 | 34.82 ± 2.94 | 305.00 ± 66.47 | 0.07 ± 0.03 | 487.80 ± 6.51 | 41.00 ± 4.24 | 27.00 ± 1.41 | 33.00 ± 2.55 |
|  | 14 | 24.16 ± 1.22 | 12.25 ± 1.63 | 39.65 ± 4.31 | 276.00 ± 28.28 | 0.33 ± 0.03 | 619.10 ± 85.56^a^ | 115.50 ± 8.41 | 30.40 ± 6.93 | 40.50 ± 4.38 |
| 25 mm | 0 | 13.76 ± 4.96 | 11.70 ± 0.14 | 37.95 ± 0.49 | 284.00 ± 53.74^a,b^ | 0.73 ± 0.17 | 235.35 ± 0.33^b,c^ | 36.30 ± 4.53^d^ | 48.65 ± 13.36 | 25.12 ± 0.03 |
|  | 1 | 26.35 ± 18.09 | 12.78 ± 1.87 | 43.05 ± 1.07 | 355.50 ± 6.36^a^ | 0.06 ± 0.00 | 505.10 ± 26.30^b^ | 38.70 ± 6.36^e^ | 37.20 ± 4.38 | 36.95 ± 11.67 |
|  | 14 | 21.90 ± 0.61 | 14.57 ± 0.61 | 49.81 ± 0.15 | 341.99 ± 0.01^b^ | 0.52 ± 0.10 | 517.35 ± 52.26^c^ | 115.44 ± 0.37^c,d,e^ | 30.11 ± 0.01 | 40.65 ± 1.20 |
| *p v*alue | L | 0.488 | 0.251 | < 0.01 | 0.429 | < 0.01 | 0.213 | 0.534 | 0.132 | 0.860 |
|  | T | 0.329 | 0.143 | < 0.01 | 0.719 | < 0.01 | < 0.05 | < 0.001 | 0.125 | < 0.05 |
|  | Interaction | 0.395 | 0.359 | 0.073 | 0.863 | < 0.01 | 0.494 | 0.384 | 0.474 | 0.764 |

Data are presented as mean ± standard deviation.

^a-e^ Superscripts indicate significant differences between the groups within the same column (*p* < 0.05; two-way ANOVA followed by Bonferroni post-hoc test).

Note. RFA, radiofrequency ablation; WBC, white blood cells; HGB, hemoglobin; HCT, hematocrit; PLT, platelet; TBIL, total bilirubin; ALP, alkaline phosphatase; AST, aspartate aminotransferase; ALT, alanine aminotransferase; GGT, gamma-glutamyl transferase.

**Supplementary Table 3.** Cholangiographic findings at baseline and immediately, 24 hours, and 2 weeks after RFA in the porcine bile duct.

| Parameter | Electrode | Baseline | Immediately | 24 h | 2 wk |
| --- | --- | --- | --- | --- | --- |
| Luminal diameter (mm) | 20 mm | 8.01 ± 0.38 | 2.25 ± 0.34^***^ | 2.18 ± 0.33^***^ | 1.99 ± 0.41^***^ |
|  | 25 mm | 7.81 ± 0.51 | 2.48 ± 0.21^***^ | 2.36 ± 0.36^***^ | 2.19 ± 0.39^***^ |
| Stricture length (mm) | 20 mm | - | 10.75 ± 0.83 | 11.50 ± 0.50 | 13.71 ± 0.71 |
|  | 25 mm | - | 16.25 ± 0.43 | 16.92 ± 0.61^†^ | 18.25 ± 0.31^†††^ |

Data are presented as mean ± standard deviation.

^***^*p* < 0.001 vs. baseline; ^†^*p* < 0.05 and ^†††^*p* < 0.001 vs. immediately after RFA (two-way ANOVA followed by Bonferroni post-hoc test).

**Supplementary Table 4.** Histological findings at 24 hours and 2 weeks after RFA.

| Parameters  (Units) | 20 mm | | | 25 mm | | | *p*-value | |
| --- | --- | --- | --- | --- | --- | --- | --- | --- |
|  | 24 h | 2 wk | 24 h | | | 2 wk | 24 h vs. 2 wk with 20-mm SES electrodes | 24 h vs. 2 wk in 25-mm SES electrodes |
| Microscopic ablation length (mm) | 12.10 ± 0.65 | 14.35 ± 0.42 | 15.61 ± 1.07 | | 18.15 ± 0.90 | | 0.001 | < 0.001 |
| Microscopic ablation depth (mm) | 2.41 ± 0.24 | 2.92 ± 0.27 | 2.62 ± 0.20 | | 2.99 ± 0.23 | | < 0.001 | < 0.001 |
| Inflammatory cell  infiltration (degree) | 4.06 ± 0.57 | 3.87 ± 0.80 | 4.37 ± 0.61 | | | 3.93 ± 0.77 | 0.454 | 0.087 |
| Collagen deposition  (degree) | 1.93 ± 0.68 | 3.68 ± 0.70 | 2.12 ± 0.71 | | | 3.62 ± 0.61 | < 0.001 | < 0.001 |
| TUNEL deposition  (degree) | 2.93 ± 0.68 | 3.50 ± 0.51 | 3.12 ± 0.80 | | | 3.75 ± 0.77 | 0.013 | 0.033 |
| HSP-70 deposition  (degree) | 4.12 ± 0.71 | 2.68 ± 0.70 | 4.06 ± 0.77 | | | 2.81 ± 0.65 | < 0.001 | < 0.001 |

Data are presented as mean ± standard deviation and compared using Mann–Whitney *U*-test.

Note. TUNEL, terminal deoxynucleotidyl transferase-mediated dUTP nick end labeling; HSP-70, heat shock protein-70.

**Supplementary Movie 1.** Procedural steps for deployment and recapture of the self-expandable stent (SES)-based endobiliary radiofrequency ablation (SES-RFA) system.
